# Interrupted time series datasets from studies investigating the impact of interventions or exposures in public health and social science: a data note

**DOI:** 10.1186/s13104-024-07055-5

**Published:** 2025-01-22

**Authors:** Simon L Turner, Elizabeth Korevaar, Amalia Karahalios, Andrew B Forbes, Joanne E McKenzie

**Affiliations:** 1 School of Public Health and Preventive Medicine, Monash University, Level 4, 553 St Kilda Road, Melbourne, VIC 3004 Australia; 2 Centre for Epidemiology and Biostatistics, Melbourne School of Population and Global Health, University of Melbourne, Melbourne, VIC 3010 Australia

**Keywords:** Interrupted time series, Meta-analysis, Public health, Empirical evaluation

## Abstract

**Objectives:**

The interrupted time series (ITS) design is commonly used to investigate the impact of an intervention or exposure in public health. There are many statistical methods that can be used to analyse ITS data and to meta-analyse their results. We undertook two empirical studies to investigate: (i) how effect estimates (and associated statistics) compared when six statistical methods were applied to 190 real-world datasets; and (ii) how meta-analysis effect estimates (and associated statistics) compared when the combinations of two ITS analysis methods and five meta-analysis methods were applied to 17 real-world meta-analyses including 283 ITS datasets. Here we present a curated repository of a subset of ITS datasets from these studies.

**Data description:**

The repository includes 430 ITS datasets curated from the two empirical studies. The datasets are diverse in the populations, interruptions and outcomes examined, and are methodologically diverse in the outcome types, aggregation time intervals, number of timepoints and segments. Most of the datasets are from public health. For each dataset, we provide the outcome value at each timepoint and the segment (indicating different interruptions), along with characteristics of the dataset. This repository may be of value for future research of ITS studies, and as a source of examples of ITS for use in teaching.

## Objective

The interrupted time series (ITS) design is commonly used to investigate the impact of population-level interventions (e.g., government policy introduction [1–3]) or exposures (e.g., natural disaster [4, 5]). In an ITS design, data are collected at multiple time points before and after an interruption, and are often aggregated using a summary statistic (e.g., mean, proportion) within a time interval (e.g., weekly, monthly) [6]. A pre-post interruption comparison is then made, while controlling for the pre-interruption trend. The design is often used when randomisation is difficult or impossible, and is less susceptible to bias compared with other non-experimental designs [6–9]. These features have led to the design’s inclusion in systematic reviews, and meta-analyses, to estimate the effects of population-level interruptions [10, 11].

Many statistical methods can be used to analyse ITS data (with the methods differing in how they account for underlying patterns in the time series data) and many methods are available to meta-analyse the resulting effect estimates. To understand whether the choice of ITS analysis method, and when meta-analysing results from ITS studies, whether the combination of ITS analysis and meta-analysis methods matters when applied to real-world datasets, we undertook two empirical evaluations [12, 13]. These empirical evaluations have companion numerical simulation studies that examine the performance of the same ITS analysis and meta-analysis methods under controlled scenarios [14, 15]. The ITS datasets were identified from two methodological reviews [10, 16]. A subset of these ITS datasets have been used to examine the accuracy of results calculated from data digitally extracted from ITS graphs [17].

We have curated the datasets from the empirical studies to form a repository including 430 ITS datasets. The datasets are diverse in the research questions addressed and in their methodological characteristics.

### Data description

#### Description of how the ITS datasets were sourced

This repository of 430 datasets has been curated from ITS datasets included in two statistical empirical studies [12, 13]. Details of the methods used to obtain the datasets from each empirical study are now outlined.

Empirical study 1 – Turner et al. [12]: We sourced 190 ITS datasets from 200 ITS studies that investigated the impact of interruptions on public health related outcomes. Full details of the methods to select studies, and the series within, are available in Turner et al. [16, 18]. We sourced ITS datasets using three methods: (i) data provided with the study; (ii) data obtained from authors via email; and, (iii) digital extraction of data from graphs provided in the published manuscripts. Digital extraction was undertaken using the software WebPlotDigitizer, which has been shown to be an accurate tool for data extraction [17, 19, 20]. This repository includes a subset of the ITS datasets sourced from methods (i) (8 datasets) and (iii) (176 datasets) above.

Empirical study 2 – Korevaar et al. [13]: We sourced 283 ITS datasets from 17 meta-analyses (included in 17 reviews investigating the impacts of, primarily, public health interruptions) that included results from at least two ITS studies. Full details of the methods used to select reviews, meta-analyses, and studies are available in Korevaar et al. [10, 21]. We sourced the ITS datasets using the same methods as for Turner et al. [16]. This repository includes a subset of the ITS datasets obtained from methods (i) (16 datasets), (ii) (220 datasets) and (iii) (10 datasets) above.

### Description of data files

The repository includes four Excel spreadsheet files (Table 1) accessible at 10.26180/24287338 [22]. A brief description of the files follows:

- Datafile 1 - (data_dictionary.xls) – is a data dictionary that describes the variables in *Datafile 2* and *Datafile 3*.
- *Datafile 2* - (study_information.xls) – is the study information file containing an indicator of the repository source (Turner et al. [12] or Korevaar et al. [13]); study citation details; study and series identification numbers that link to the ITS datasets (in *Datafile 3*); description and type of the intervention; description, direction of benefit and type of the outcome (e.g., rate); length of the time series and time interval (e.g., 12 datapoints of monthly data); and, which method was used to source the ITS dataset.
- *Datafile 3* - (time_series_data.xls) - is the time series data file containing an indicator of the repository source (Turner et al. [12] or Korevaar et al. [13]); study and series identification numbers (i.e. linking variables with *Datafile 2)*; time variable; numerical value of the outcome; segment (1 for the first segment, 2 for the second, etc.); and, an indicator of whether the datapoints were outliers or part of a transition period.
- *Datafile 4* - (version_history.xls) – this file has been added in anticipation of further data being added to the repository and will include a description of changes made between versions of the repository as new datasets are added.

If you wish to contribute ITS datasets to the repository, please contact the corresponding author.

**Table 1:** Overview of data files

Label	Name of data file/data set	File types (file extension)	Data repository and identifier (DOI or accession number)
Datafile 1: Data dictionary	data_dictionary.xls	MS Excel file (.xlsx)	Monash University (10.26180/24287338) (22)
Datafile 2: Summary study description	study_information.xls	MS Excel file (.xlsx)	Monash University (10.26180/24287338) (22)
Datafile 3: Time series data	time_series_data.xls	MS Excel file (.xlsx)	Monash University (10.26180/24287338) (22)
Datafile 4: Version description	version_history.xls	MS Excel file (.xlsx)	Monash University (10.26180/24287338) (22)

### Limitations

We could not obtain data for all the eligible time series included in the reviews [10, 16]. There are additional limitations which vary by the methods used to obtain the data. For data sourced as part of the Turner et al. [12, 16] studies, limitations include:

- The majority of the data series were obtained using digital data extraction, and could contain errors (though these are unlikely to result in any substantive errors [17]).
- Due to difficulty digitally extracting data from particularly long data series not all of the longer series identified in the review [16] are part of this repository.

For data sourced as part of the Korevaar et al. [10, 13] studies, limitations include:

- The descriptions of the outcomes and interventions were extracted from the reviews, and not the primary studies. Therefore, any inaccuracy in the description of these variables in the review, will also be inaccurate in our repository.

Furthermore, our repository does not capture all details of the ITS datasets. For example, we have only captured the description of the first interruption, but not captured any descriptions of the pre-interruption, or potentially, other interruption segments; nor have we captured details of when and where the studies were undertaken. However, for those requiring further detail, citations are provided to the primary studies so that further information can be extracted if required.

## Data Availability

The data described in this data note can be freely and openly accessed on the Monash University repository known as Bridges, https://doi.org/10.26180/24287338. Please see Table 1 and reference (22) for details and links to the data.

## Abbreviations

ITS: Interrupted Time Series
